# Real‐time automatic image‐based slice tracking of gadolinium‐filled balloon wedge catheter during MR‐guided cardiac catheterization: A proof‐of‐concept study

**DOI:** 10.1002/mrm.29822

**Published:** 2023-09-07

**Authors:** Rohini Vidya Shankar, Li Huang, Radhouene Neji, Grzegorz Kowalik, Alexander Paul Neofytou, Ronald Mooiweer, Tracy Moon, Nina Mellor, Reza Razavi, Kuberan Pushparajah, Sébastien Roujol

**Affiliations:** ^1^ Biomedical Engineering Department, School of Biomedical Engineering and Imaging Sciences King's College London London UK; ^2^ MR Research Collaborations, Siemens Healthcare Limited Camberley UK; ^3^ Guy's and St Thomas' NHS Foundation Trust London UK

**Keywords:** cardiac catheterization, image processing, MR guidance, partial saturation, passive tracking, real time

## Abstract

**Purpose:**

MR‐guided cardiac catheterization procedures currently use passive tracking approaches to follow a gadolinium‐filled catheter balloon during catheter navigation. This requires frequent manual tracking and repositioning of the imaging slice during navigation. In this study, a novel framework for automatic real‐time catheter tracking during MR‐guided cardiac catheterization is presented.

**Methods:**

The proposed framework includes two imaging modes (*Calibration* and *Runtime*). The sequence starts in *Calibration* mode, in which the 3D catheter coordinates are determined using a stack of 10–20 contiguous saturated slices combined with real‐time image processing. The sequence then automatically switches to *Runtime* mode, where three contiguous slices (acquired with partial saturation), initially centered on the catheter balloon using the *Calibration* feedback, are acquired continuously. The 3D catheter balloon coordinates are estimated in real time from each *Runtime* slice stack using image processing. Each *Runtime* stack is repositioned to maintain the catheter balloon in the central slice based on the prior *Runtime* feedback. The sequence switches back to *Calibration* mode if the catheter is not detected. This framework was evaluated in a heart phantom and 3 patients undergoing MR‐guided cardiac catheterization. Catheter detection accuracy and rate of catheter visibility were evaluated.

**Results:**

The automatic detection accuracy for the catheter balloon during the *Calibration/Runtime* mode was 100%/95% in phantom and 100%/97 ± 3% in patients. During *Runtime*, the catheter was visible in 82% and 98 ± 2% of the real‐time measurements in the phantom and patients, respectively.

**Conclusion:**

The proposed framework enabled real‐time continuous automatic tracking of a gadolinium‐filled catheter balloon during MR‐guided cardiac catheterization.

## 1 INTRODUCTION

Diagnostic and interventional cardiac catheterization procedures guided by X‐ray fluoroscopy are routinely performed in patients with congenital heart disease (CHD), leading to improved outcomes and increased life expectancy.^1^, ^2^, ^3^ During these procedures, catheters are navigated through the cardiovascular system and used for interventional purposes (such as balloon dilation) or for diagnostic purposes (pressure measurements). Guidewires can also be used to facilitate placement of the catheter. MRI is an attractive alternative to X‐ray fluoroscopy for the guidance of cardiac catheterization procedures,^4^, ^5^, ^6^, ^7^, ^8^, ^9^, ^10^, ^11^, ^12^, ^13^, ^14^, ^15^, ^16^ as it not only avoids exposure to harmful ionizing radiation especially undesirable in young patients undergoing repeat procedures,^17^, ^18^ but also offers high soft‐tissue contrast, superior hemodynamic data, easy manipulation of the imaging planes, and real‐time 3D acquisition and reconstruction of complex anatomy.^11^, ^19^, ^20^, ^21^

Passive tracking approaches are currently used clinically for real‐time visualization of the catheter during MR guidance.^4^, ^5^, ^6^, ^8^, ^9^, ^10^, ^11^, ^12^, ^14^, ^15^, ^16^, ^22^ These approaches commonly use balloon wedge catheters, which can be filled with CO_2_ for negative contrast visualization (hypo‐intense signal) or diluted Gadolinium (Gd) for positive contrast visualization (hyperintense signal) in the images. Gd‐filled balloon wedge catheters tend to be more conspicuous and faster to navigate compared with air‐filled balloons.^9^ Various magnetization preparation schemes have been used for improved visualization of the Gd‐filled catheter balloon. A saturation prepulse that can be turned on to visualize the balloon and turned off to visualize the soft tissues and blood was proposed.^9^ Black blood preparation with flow‐sensitive gradients was used for simultaneous visualization of the catheter balloon and soft tissues.^23^ A saturation prepulse with a reduced saturation angle to achieve partial saturation (pSAT) preparation was proposed for simultaneous high‐contrast visualization of the catheter balloon, soft tissues, and blood.^15^ This technique offered excellent visualization capabilities during MR‐guided cardiac catheterization in CHD patients.^16^

All passive tracking approaches that are used clinically require frequent manual slice tracking and manipulation of the imaging plane to follow the balloon during catheter navigation. This is usually achieved by the interventionist using foot pedals or via the scanner console. In a recent study, it was reported that the catheter fell out of plane in more than 30% of real‐time measurement frames during navigation.^16^ This diverts the focus of the operator from the catheter navigation and prolongs the intervention, reducing the value of image guidance.

A T_1_ overlay method in which brighter signals from T_1_‐weighted images were overlaid to 3D multiplanar reconstruction views increased the catheter visualization time to 90%.^6^ This study used a thick 20‐mm slice, which could partly explain the reduced out‐of‐plane time of the catheter; however, this could also potentially reduce the value of the images in narrower and complex anatomies. Furthermore, physiological motion and any deformation induced by the interventional device would not be captured using a 3D visualization from a static volume, which could result in the misregistration of the real‐time balloon signal relative to the underlying anatomical roadmap.

In this study, we present a novel framework that enables real‐time image‐based tracking of the catheter balloon and automatic repositioning of the imaging slice (i.e., slice tracking) for continuous high‐contrast visualization of the balloon and anatomy during catheter navigation. We demonstrate the proposed technique in a phantom and subsequently present its feasibility in patients undergoing MR‐guided cardiac catheterization. Part of this work has previously been presented as conference proceedings.^24^, ^25^

## 2 METHODS

### 2.1 Proposed framework

**FIGURE 1:**
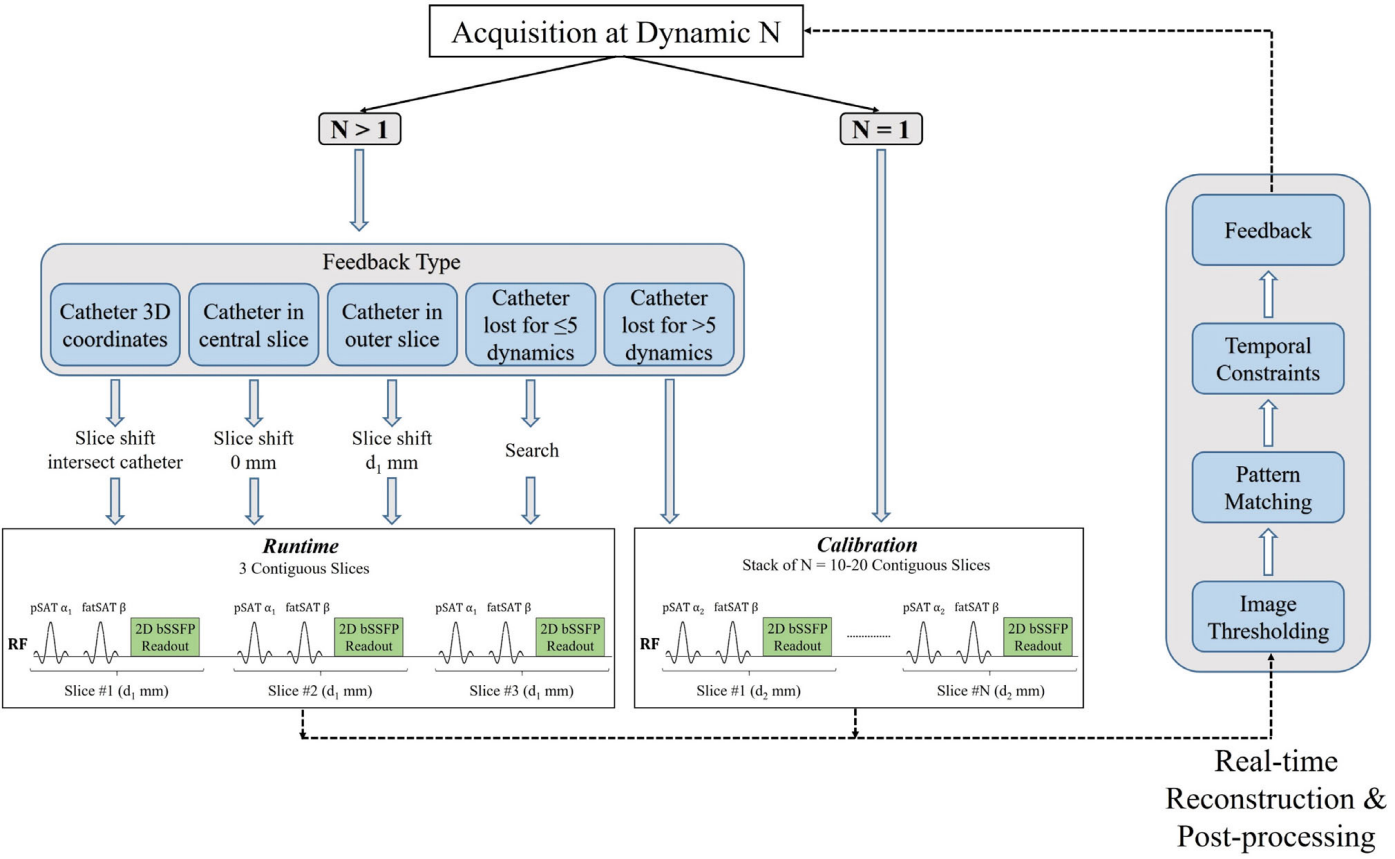
The proposed framework illustrating the acquisition/imaging modes and image‐processing steps for real‐time automatic tracking of the catheter balloon. Here, d_1_ = d_2_ = 10 mm, partial saturation (pSAT) α_1_ = 30^∘^–50^∘^, pSAT α_2_ = 90^∘^, and fatSAT β = 90^∘^.

The proposed prototype sequence consists of two imaging modes: *Calibration* and *Runtime* (Figure 1 **)**. To achieve optimal contrast between the catheter balloon and surrounding anatomy, all image acquisitions are preceded by nonselective pSAT and chemical shift–selective fat‐suppression pulses. The sequence begins with the *Calibration* mode in which a fixed stack of contiguous slices (n = 10–20, slice thickness = 10 mm, pSAT = 90^0^) is acquired in under 3 s. Real‐time image processing of this slice stack (described in Section 2.2) is performed to identify the initial 3D coordinates of the balloon without any prior knowledge of its location. The sequence then automatically switches to the *Runtime* mode, where three contiguous slices in the orientation of interest (slice thickness = 10 mm, pSAT = 30^∘^–50°, adjustable via the scanner console) are acquired continuously. A pSAT of 30^∘^–50° has previously been shown to provide a good compromise between visualization of the cardiovascular anatomy and balloon/blood contrast.^15^ Initially, based on the 3D coordinates obtained from the *Calibration* mode, the first three *Runtime* slices are automatically positioned to intersect the balloon in the central slice. During *Runtime*, the 3D balloon position is continuously estimated from real‐time image processing of the three slices (detailed in Section 2.2). If the balloon is detected in either of the outer slices, the three slices are automatically repositioned to ensure that the catheter is in the central slice (i.e., the *Runtime* stack is shifted by one slice thickness toward the outer slice containing the catheter balloon). Furthermore, the sequence automatically switches back to the *Calibration* mode if the balloon is lost for more than 3 s (i.e., > 5 real‐time measurements, adjustable via the scanner console), such as when the catheter leaps beyond the three‐slice through‐plane range in the *Runtime* mode. The different feedback scenarios are shown in Figure 1.

The proposed sequence and postprocessing were developed within the manufacturer's programming environments to enable online real‐time acquisition and tracking. Images were visualized using a standard commercially available inline display window on the scanner console. This screen was mirrored onto an LCD monitor located inside the scanner room to provide the same view to the interventionist.

### 2.2 Real‐time image processing

Each stack of images acquired during the *Calibration* and *Runtime* modes is processed using the following steps, unless specified otherwise (the corresponding pseudo code is provided in Script S1 in Data S1).

1. First, the image stack is binarized using a signal intensity threshold to identify hyperintense pixels and generate a binary image stack. Two different thresholds are used for the *Calibration* and *Runtime* modes (90% and 40% of the maximum signal intensity of the stack, respectively, adjustable via the scanner console). This processing is restricted to the volume of interest corresponding to the prescribed shim box encompassing the entire cardiovascular system navigated during the intervention to eliminate any spurious signals from the edge of the FOV.
2. Morphological closing, which involves the processes of dilation followed by erosion (using a 3 × 3 square structuring element), is performed on this binary image stack to fill/merge any small holes into the background.
3. All detected pixels are clustered into multiple contiguous regions.
4. Any region with a large area (> disc area with an 8‐mm radius) or a maximum distance to its center of mass > 8 mm is then discarded. Note that 8 mm was selected as an upper bound of the balloon radius and is adjustable via the scanner console.
5. Additionally, the catheter displacement is expected to show some temporal consistency between consecutives frames. To account for this, in the *Runtime* mode, regions that are considered too distant (using a maximum distance threshold empirically set to 14 mm, adjustable via the scanner console) from the previous catheter position are discarded.
6. For each slice, if multiple regions remain, the region closest to the previous catheter location is selected in that slice.
7. If multiple regions are identified across all slices, the region with the brightest signal is selected as the balloon and its 3D coordinates are computed.

The user‐defined parameters used in this real‐time image processing pipeline were optimized in a phantom experiment presented in Tables S1–S4 in Data S1.

### 2.3 Experimental evaluation

All imaging experiments were performed on a 1.5T MRI scanner (MAGNETOM Aera; Siemens Healthcare, Erlangen, Germany). The balloon of the wedge catheter (Arrow; Teleflex, Wayne, PA, USA) was filled with 1% Gd (Dotarem; Guerbet, Villepint, France) for positive contrast visualization in all the experiments. This study was approved by the local institutional review board (REC reference: 21/LO/0650 IRAS project ID: 304329).

#### 2.3.1 Phantom experiment

The proposed framework was tested in a 3D‐printed heart phantom, which was printed from the segmented anatomy of a healthy adult subject obtained using a high‐resolution MRI scan. Both the *Calibration* and *Runtime* modes used a 2D single‐shot acquisition with the same balanced SSFP readout and the following parameters: TR/TE = 2.44/0.99 ms, flip angle (FA) = 50°, FOV = 450 × 450 mm^2^, reconstructed resolution = 2.8 × 2.8 mm^2^, slice thickness = 10 mm, temporal resolution = 209.4 ms, number of real‐time measurements = 99, bandwidth = 1010 Hz/px, GRAPPA factor = 2, partial Fourier = 5/8. The *Calibration* stack was prescribed in the coronal orientation and remained fixed during the procedure. The *Runtime* slices were prescribed along the coronal orientation. The catheter was manipulated through the phantom during the entire acquisition. In two instances, the catheter was deliberately moved rapidly in the through‐plane direction to force the loss of the balloon from the *Runtime* slices during navigation.

#### 2.3.2 Evaluation in patients

The in vivo feasibility of the proposed approach was investigated in 3 CHD patients (10 ± 3 years, all male, weight = 31 ± 10 kg) undergoing MR‐guided cardiac catheterization. The 2D single‐shot balanced SSFP acquisition parameters were the same as those for the phantom experiment except for the following: the number of real‐time measurements was 19, 29, and 59 for Patients 1, 2, and 3, respectively. The sequence was run during catheter manipulation. The *Calibration* stack was prescribed in the coronal orientation for fast screening of the cardiovascular system because the anterior/posterior usually represents the smaller body dimension and remained fixed during the procedure. The *Runtime* slices were prescribed in the sagittal direction in all 3 patients.

### 2.4 Analysis

The accuracy of automatic identification of the catheter balloon was assessed in the phantom and patients. We verified whether the computed catheter coordinates matched the actual location of the catheter balloon in the magnitude images. A correct catheter detection was defined as the center of mass of the estimated balloon region falling within the true region of the balloon. Accuracy was determined for both the *Calibration* and *Runtime* modes and expressed as a percentage of the total number of acquired real‐time measurements. For the *Runtime* mode, we also examined the percentage of true positive (balloon present and correctly detected), true negative (balloon absent and not detected), false positive (balloon absent and detected), and false negative (balloon present and not detected) outcomes for catheter detection of the real‐time image processing detailed earlier. The percentage of time the sequence was in the *Calibration* and *Runtime* modes was calculated. Furthermore, we computed the percentage of real‐time measurements the catheter balloon was visible in the magnitude images in the *Runtime* mode during catheter navigation.

## 3 RESULTS

### 3.1 Phantom experiment

**FIGURE 2:**
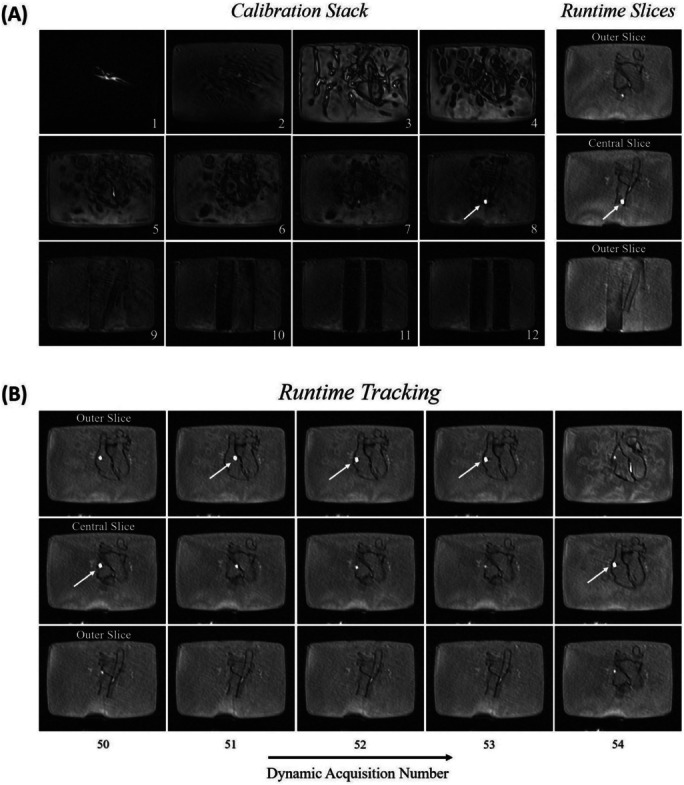
Three‐dimensional printed heart phantom experiment. (A) *Calibration* stack (12 slices) with the balloon initially identified in Slice 8, with subsequent automatic switch to the *Runtime* mode with the three slices correctly centered on the balloon. (B) *Runtime* slices acquired at later real‐time measurements, demonstrating automatic slice following (between real‐time measurements 53 and 54) when the balloon was detected in one of the outer slices. The white arrows indicate the position of the automatically identified balloon.

Figure 2 shows still frames of the phantom experiment. A movie of the experiment is shown in Video S1. The balloon was initially detected in the first *Calibration* stack of the acquisition (Slice 8). The 3D balloon coordinates obtained via feedback from the *Calibration* mode were then used to automatically position the three *Runtime* slices to intersect the balloon in the central slice (Figure 2A). Selected still frames acquired during *Runtime* while the catheter was navigated are shown in Figure 2B. This set of *Runtime* slices demonstrates automatic slice following and repositioning (between real‐time measurements 53 and 54), performed to continuously intersect the balloon in the central slice. Video S1 also shows the scenario in which, after fast displacement of the catheter, the balloon fell out of plane from the three *Runtime* slices. This triggered an automatic switch to the *Calibration* mode for quick redetection of the catheter and repositioning of the *Runtime* slices (real‐time measurements 75–88).

The balloon was automatically identified with 100% accuracy whenever the sequence was in the *Calibration* mode. In the *Runtime* mode, the detection accuracy across the three‐slice stack was 95%. Furthermore, the percentage of true positive, true negative, false positive, and false negative outcomes were 77%, 18%, 0%, and 5%, respectively. During the experiment, the sequence was in the *Runtime* mode for 97% of the time, with the remaining time in the *Calibration* mode. The balloon was visible in the magnitude images in 82% of all real‐time measurements during the *Runtime* mode. The remaining 18% of real‐time measurements correspond to the part of the experiment when the balloon was briefly lost due to deliberate fast displacement of the catheter beyond the three‐slice through‐plane range.

### 3.2 Feasibility in patients

**FIGURE 3:**
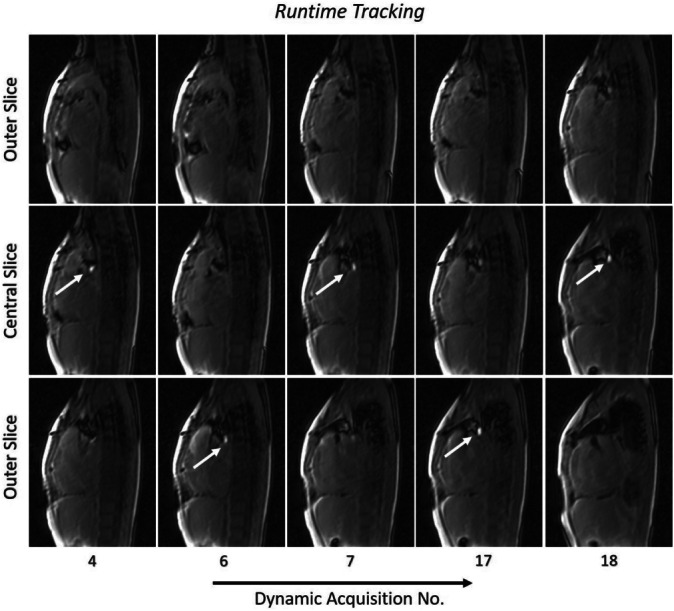
Representative *Runtime* images demonstrating the proposed approach in Patient 1. Automatic slice tracking and repositioning (between real‐time measurements 6 and 7 as well as 17 and 18) were performed when the balloon was detected in one of the outer slices. The white arrows indicate the location of the automatically identified balloon.

**FIGURE 4:**
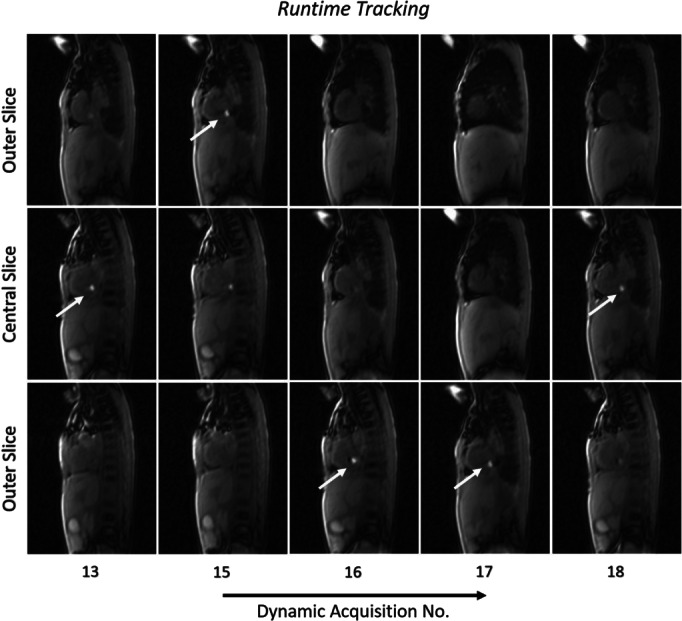
*Runtime* slices illustrating the proposed approach in Patient 2. The three‐slice stack was automatically adjusted to ensure balloon visibility and follow the catheter in the central slice. The white arrows show the detected location of the catheter balloon.

Figures 3 and 4 along with Figures S1 and S2 in Data S1 show the application of the proposed sequence in Patients 1 and 2 undergoing MR‐guided cardiac catheterization. The catheter balloon was detected in the *Calibration* stack (Slice 7 in Patient 1 and Slice 6 in Patient 2), followed by automatic centering of the *Runtime* slices on the balloon. Catheter tracking with slice repositioning during *Runtime* was then successfully achieved while the catheter was manipulated. Video S2 shows a movie of the in vivo tracking in Patient 1. When the balloon was identified in one of the outer slices, the *Runtime* slices were automatically shifted (between real‐time measurements 6 and 7 and 17 and 18) to intersect the catheter in the central slice. Video S3 shows another movie of the automatic tracking in Patient 3.

In all 3 patients, the balloon was automatically identified with 100% accuracy when the sequence was in the *Calibration* mode. During *Runtime*, the detection accuracy was 97 ± 3%. During the in vivo procedures, the sequence was in the *Runtime* mode for 95%, 97%, and 98% of the time for Patients 1–3, respectively, with the remaining corresponding times in the *Calibration* mode. The balloon was clearly visible in the magnitude images during *Runtime* in 100%, 96%, and 98% of the real‐time measurements in Patients 1–3, respectively. Additionally, the SNR values for the balloon and blood, and contrast‐to‐noise ratio (CNR) for balloon/blood for the 3 patients, were SNR_balloon_ = 81 ± 2, SNR_blood_ = 28 ± 2, and CNR_balloon/blood_ = 52 ± 3. The results for the phantom and patients are summarized in Table 1.

**TABLE 1:** The accuracy of automatic detection of the catheter balloon and rate of catheter visibility in the phantom and patients, along with the SNR and contrast‐to‐noise ratio (CNR) values of the balloon/blood in vivo.

	Automatic Detection Accuracy						
	*Calibration*	*Runtime*	Rate of Catheter Visibility^a^	Number of Real‐Time Measurements	SNR (balloon)	SNR (blood)	CNR (balloon/blood)
Phantom	100%	95%	82%^b^	99	—	—	—
Patient 1	100%	94%	100%	19	83	29	54
Patient 2	100%	100%	96%	29	79	30	49
Patient 3	100%	97%	98%	59	80	26	54

^a^ In the magnitude images (*Runtime*).

^b^ In two instances, the catheter was deliberately moved rapidly in the through‐plane direction to force the loss of the balloon from the *Runtime* slices during navigation.

The computation time of the proposed real‐time postprocessing was 20 ms for a stack of three *Runtime* slices, and the latency, defined as the reconstruction time of the three *Runtime* slices (˜200 ms/slice), postprocessing, and feedback sent and received by the acquisition process, was 650 ms.

## 4 DISCUSSION

In this study, we developed a novel cardiac MRI sequence that enables automatic real‐time tracking and visualization of a Gd‐filled balloon during catheter navigation. The proposed acquisition and image processing framework incorporates (i) partial saturation with high spatial coverage, (ii) automatic image‐based estimation of the catheter balloon position, and (iii) real‐time slice following and repositioning. This framework was successfully demonstrated in a 3D‐printed heart phantom, and its in vivo feasibility was shown in 3 patients. The sequence achieved automatic continuous visualization of the balloon with high detection accuracy, and improved visibility of the catheter was also achieved compared with existing approaches. Furthermore, the SNR and CNR values for the balloon/blood in vivo were found to be comparable with the values reported in a previous pSAT study in patients.^16^

The image processing of the slice stacks in both imaging modes was based on several user‐defined parameters. Catheter detection was restricted to the volume of interest covered by the prescribed shim box, which was implemented to reduce the identification of false/spurious structures (especially fat signal) at the edges of the FOV. The prescription of a region of interest encompassing the entire cardiovascular system and excluding bright structures (such as fat signal from the chest and back) may be challenging in certain patients using a rectangular box. Nevertheless, fat signal areas from the chest and back have a different spatial pattern than the catheter balloon and may be robustly discarded using these spatial pattern constraints. The need for a more robust rejection algorithm, such as using a nonlinear region of interest around the targeted anatomy, requires further investigation. Furthermore, spatiotemporal constraints were added to restrain possible solutions based on the expected catheter movement within the imaging interval. These parameters were suitable for the described experiments but may require adjustments to ensure the robustness of the framework in a larger patient cohort.

The proposed sequence can facilitate the fast detection of out‐of‐plane catheters, which are time‐consuming to locate when manually tracked and can prolong the procedure. When the catheter suddenly moved beyond the three‐slice through‐plane range in the *Runtime* mode, a controllable lost limit ensured an automatic switch back to *Calibration* mode for re‐estimation of the balloon coordinates within the prescribed *Calibration* stack. However, it may be desirable to introduce the possibility for the operator to manually switch to the *Calibration* mode, such as by using a pedal. This could be useful in cases in which the operator judges that the catheter may come back in‐plane shortly or if the sequence identifies a different structure as the catheter. Although the latter case was not observed in our study, this could in theory occasionally happen when navigating the catheter in the vicinity of vessels depicting bright in‐flow artifacts or small fat areas that could be mistaken for the catheter.

The *Runtime* mode is based on the real‐time sequential acquisition of three contiguous slices, resulting in reduced temporal resolution compared with single‐slice imaging. This can be avoided by using a single slice only during *Runtime* at the cost of a more frequent switch back to the *Calibration* mode (as demonstrated in the Supporting Information and corresponding Videos S4 and S5) or using accelerated imaging schemes such as simultaneous multislice imaging.^26^ Achieving a higher framerate may also facilitate the acquisition of orthogonal orientations during the *Runtime* mode, which may provide useful additional anatomical context to the user.^10^, ^16^ Additionally, the availability of simultaneous multiple views may potentially improve the performance of the catheter balloon detection and tracking. Furthermore, all three slices have the same slice thickness. The use of larger slice thicknesses combined with higher pSAT angles for the two outer slices could be investigated to further reduce the likelihood of losing the catheter during *Runtime*.

The proposed approach was evaluated at 1.5 T using a Gd‐filled catheter balloon. Despite reduced conspicuity, the use of a gas‐filled catheter balloon may have benefits for MRI‐guided cardiac catheterizations due to improved buoyancy.^27^ The current postprocessing pipeline would need adjustment for tracking of hypo‐intense signal in the images, which will be the focus of future work. Furthermore, the use of low‐field MRI scanners may be attractive for this application due to larger bore sizes facilitating patient access and catheter manipulation particularly in small patients. It may also offer the possibility to use off‐the‐shelf devices with better mechanical properties than MR‐conditional ones.^28^ Evaluation of the proposed framework at low field will also be investigated in the future.

This study has some limitations. First, the accuracy of the computed 3D balloon coordinates depends on the selection of several user‐defined parameters in the postprocessing. Although most of these parameters were kept constant in our experiments, a parameter‐free approach could further improve the robustness of the technique and facilitate its application in a clinical setting. The use of deep learning approaches may prove useful in this context, in which a neural network could be trained for image‐based detection of the catheter balloon, which could improve the robustness of the proposed technique and reduce its dependency on user‐defined parameters.^29^ Second, in this proof‐of‐concept study, the proposed framework was evaluated in only 3 patients. Further clinical evaluation in a larger patient cohort during an entire catheterization procedure and comparison with a standard approach is required to establish its advantages, especially for potentially reducing the out‐of‐plane time of the catheter and overall procedure time.

## 5 CONCLUSIONS

A novel framework was developed for real‐time automatic catheter tracking during MR‐guided cardiac catheterization. This technique enabled robust automatic tracking of the catheter during navigation while simultaneously providing high‐contrast visualization of the catheter and cardiovascular system.

## CONFLICT OF INTEREST STATEMENT

Dr. Radhouene Neji is an employee of Siemens Healthcare Limited, Camberley, United Kingdom. Dr. Li Huang is an employee of Neoscan Solutions GmbH, Magdeburg, Germany. Dr. Ronald Mooiweer is seconded to Siemens Healthcare Limited, Camberley, United Kingdom.

## Supporting information


**Video S1.** Movie of the 3D‐printed heart phantom experiment in which the gadolinium (Gd)‐filled balloon was continuously tracked while the catheter was navigated through the phantom. The white arrows indicate the location of the automatically detected catheter balloon.

**Video S2.** Movie showing the application of the proposed sequence in vivo in Patient 1. The catheter balloon was successfully identified in the *Calibration* stack (Slice 7). During *Runtime*, the three slices were automatically tracked and repositioned to ensure continuous visualization of the balloon and to track the catheter in the central slice. The white arrows indicate the automatically identified location of the balloon.

**Video S3.** Movie demonstrating the proposed automatic catheter tracking in vivo in Patient 3. The catheter balloon was initially detected in the *Calibration* stack (Slice 2). The three slices were automatically repositioned during the *Runtime* mode to intersect the catheter in the central slice. The white arrows show the automatically detected location of the balloon.

**Video S4.** Movie showing the idea of using a single slice in the *Runtime* mode in a 3D‐printed heart phantom. It can be seen that the catheter falls more frequently out‐of‐plane, followed by a more frequent switch to the *Calibration* mode.

**Video S5.** Movie showing the use of the proposed three‐slice stack in the *Runtime* mode in a 3D‐printed heart phantom. For similar navigation of the catheter through the phantom by the interventionist, as in the single‐slice experiment, the catheter was always visible in one of the three slices during *Runtime*, accompanied by frequent slice repositioning to maintain the balloon in the central slice.

**Data S1.** Supporting information.
