# Supplementary material for: Real‐time automatic image‐based slice tracking of gadolinium‐filled balloon wedge catheter during MR‐guided cardiac catheterization: A proof‐of‐concept study
Source: Magn Reson Med. 2023 Sep 7;91(1):388–97. doi: 10.1002/mrm.29822 (PMC10952810; doi:10.1002/mrm.29822)
Supplement: Supplementary file 6 — Data S1. Supporting information. [file MRM-91-388-s003.docx]

**Supplementary Information**

**(1) Supplementary Information Script S1**

Real-time image processing is performed to determine the location of the catheter balloon using the following steps:

**// Step 1) Image binarization**

Find maximum signal intensity of the entire stack

Define signal intensity threshold

Apply signal intensity thresholding to generate a binary stack

Apply dilation operator

Apply erosion operator

**// Step 2) Clustering and segmentation**

while all segmented pixels (equal to 1 in the binary stack) have not been assigned to a region

{

Select one non-assigned pixel as seed pixel

Create a new region

Apply region growing from this seed pixel and cluster pixels into the new region

}

**// Step 3) Region filtering**

for each region

{

Discard large regions

Discard non circular regions

Discard regions too far from the previous estimate

}

for each slice from the stack

{

if (multiple regions detected in slice)

{

Select region closest to previous estimate and discard remaining regions

}

**}**

if (multiple regions detected across all slices)

{

Select region with brightest signal

}

if (one region detected across the stack)

{

Compute 3D catheter balloon coordinates from center of mass of remaining region

}

else

{

No detection

}

**(2) Optimization of the user-defined parameters of the real-time image processing pipeline**

The effect of the empirical parameters used in the image processing of the *Runtime* and *Calibration* slice stacks was evaluated in a phantom experiment and each parameter is discussed in detail below:

1) Intensity thresholds used in the *Calibration* and *Runtime* modes: Two different thresholds (expressed as a % of the maximum signal intensity of the stack) are used for the *Calibration* and *Runtime* modes, which can both be adjusted via the scanner console. The influence of these thresholds on the accuracy of the catheter detection are shown below for both the *Calibration* mode (**Supplementary Information Table S1**) and *Runtime* mode (**Supplementary Information Table S2**).

**Supplementary Information Table S1:** Accuracy of automatic detection of the catheter balloon for different signal thresholding levels applied to the *Calibration* slice stack (for a balloon radius of 6 pixels (8.4 mm) and temporal distance of 10 pixels (14 mm), resolution = 1.4 x 1.4 mm^2^/pixel).

|  | Signal Intensity Threshold (*Calibration*) | | | | | | | | |
| --- | --- | --- | --- | --- | --- | --- | --- | --- | --- |
|  | 10% | 20% | 30% | 40% | 50% | 60% | 70% | 80% | 90% |
| Accuracy of Automatic Detection (%) | 33.3 | 100 | 100 | 100 | 100 | 100 | 100 | 100 | 100 |

**Supplementary Information Table S2:** Accuracy of automatic detection of the catheter balloon for different signal thresholding levels applied to the three-slice stack in the *Runtime* mode (for a balloon radius of 6 pixels (8.4 mm) and temporal distance of 10 pixels (14 mm), resolution = 1.4 x 1.4 mm^2^/pixel).

|  | Signal Intensity Threshold (*Runtime*) | | | | | | | |
| --- | --- | --- | --- | --- | --- | --- | --- | --- |
|  | 10% | 20% | 30% | 40% | 50% | 60% | 70% | 80% |
| Accuracy of Automatic Detection (%) | 8.3 | 93.8 | 96.9 | 96.9 | 96.9 | 92.7 | 62.5 | 32.3 |

The above tables show that low threshold values will increase the rate of false positives, as expected. This effect is expected to be even more pronounced in vivo due to the presence of other bright signals such as fat signal and flow artifacts, which are not visible in a phantom. For the *Runtime* mode, where a lower pSAT angle is used, higher threshold values lead to reduced accuracy of catheter detection and a compromise for the threshold selection is therefore needed. Based on these results, we decided to select thresholds of 90% and 40% for the *Calibration* and *Runtime* modes, respectively.

2) Balloon radius: This parameter is used to discard any regions that are larger than the expected area/size of the balloon and can be adjusted via the scanner interface. We found the upper bound of 8 mm to be optimal for our study, i.e., regions with a maximum distance to their centre of mass > 8 mm were discarded, which is in-line with the expected maximum size of the balloon. Nevertheless, we evaluated the effect of different balloon radii (used to calculate the expected area of the balloon) on the accuracy of automatic detection of the balloon, as shown in **Supplementary Information Table S3** below:

**Supplementary Information Table S3:** Accuracy of automatic detection of the catheter balloon for different balloon radii (for a threshold of 40% and temporal distance of 10 pixels (14 mm), resolution = 1.4 x 1.4 mm^2^/pixel).

|  | Balloon Radius* | | | |
| --- | --- | --- | --- | --- |
|  | 3 pixels  (4.2 mm) | 4 pixels  (5.6 mm) | 5 pixels  (7 mm) | 6 pixels  (8.4 mm) |
| Accuracy of Automatic Detection (%) | 13.5 | 30.2 | 96.9 | 96.9 |

*resolution = 1.4 x 1.4 mm^2^/pixel

The above results indicate that if the balloon radius is set too small, then the regions representing the balloon will be discarded. Thus, the radius should be set slightly larger than the theoretical/actual balloon radius to correctly represent its actual area. As shown in this experiment, a radius of 7-8.4 mm was found to be optimal. A threshold of 8.4 mm was selected in this study to provide some safety margin.

3) Temporal distance: In addition to the spatial constraints, a temporal constraint is applied to discard regions that are too far from the previous balloon location (adjustable via the scanner interface). This depends on the speed of movement of the catheter, where some temporal consistency is expected between consecutive frames. However, we do acknowledge that the speed of navigation may not be consistent throughout the catheterization procedure and would require a more robust metric. The effect of different temporal distances on the accuracy of automatic detection of the balloon is shown in **Supplementary Information** **Table S4** below:

**Supplementary Information Table S4:** Accuracy of automatic detection of the catheter balloon for different temporal distances (for a threshold of 40% and balloon radius of 6 pixels (8.4 mm), resolution = 1.4 x 1.4 mm^2^/pixel).

|  | Temporal Distance* | | | | |
| --- | --- | --- | --- | --- | --- |
|  | 4 pixels (5.6 mm) | 6 pixels (8.4 mm) | 8 pixels (11.2 mm) | 10 pixels (14 mm) | 12 pixels (16.8 mm) |
| Accuracy of Automatic Detection (%) | 39.6 | 77.1 | 86.5 | 96.9 | 100 |

*resolution = 1.4 x 1.4 mm^2^/pixel

The above results indicate that if a too small temporal distance is chosen, the algorithm will fail to detect the catheter if the actual displacement/navigation is not slow enough. If the temporal distance is too large and the corresponding speed of navigation is not fast enough, this might also result in false positive detections that are actually much further away from the catheter. As shown in this experiment, a choice of 10-12 pixels (14-16.8 mm), appears to offer a good compromise between the above two scenarios of catheter displacement. In this study, a temporal distance of 10 pixels was selected to balance both effects.

4) Lost limit: A controllable lost limit is used to detect out-of-plane catheters, where the sequence automatically switches to the *Calibration* mode when the catheter is lost. In this study, this was set to >5 real-time measurements (>3 s) and can be adjusted via the scanner interface. The choice of this parameter is subjective and largely depends on the skill or preference of the operator. In this study, the selection of this parameter was rather empirical and further clinical evaluation will be needed to optimize this parameter based on operator feedback.

**(3) Comprehensive representations of Figures 3 and 4**


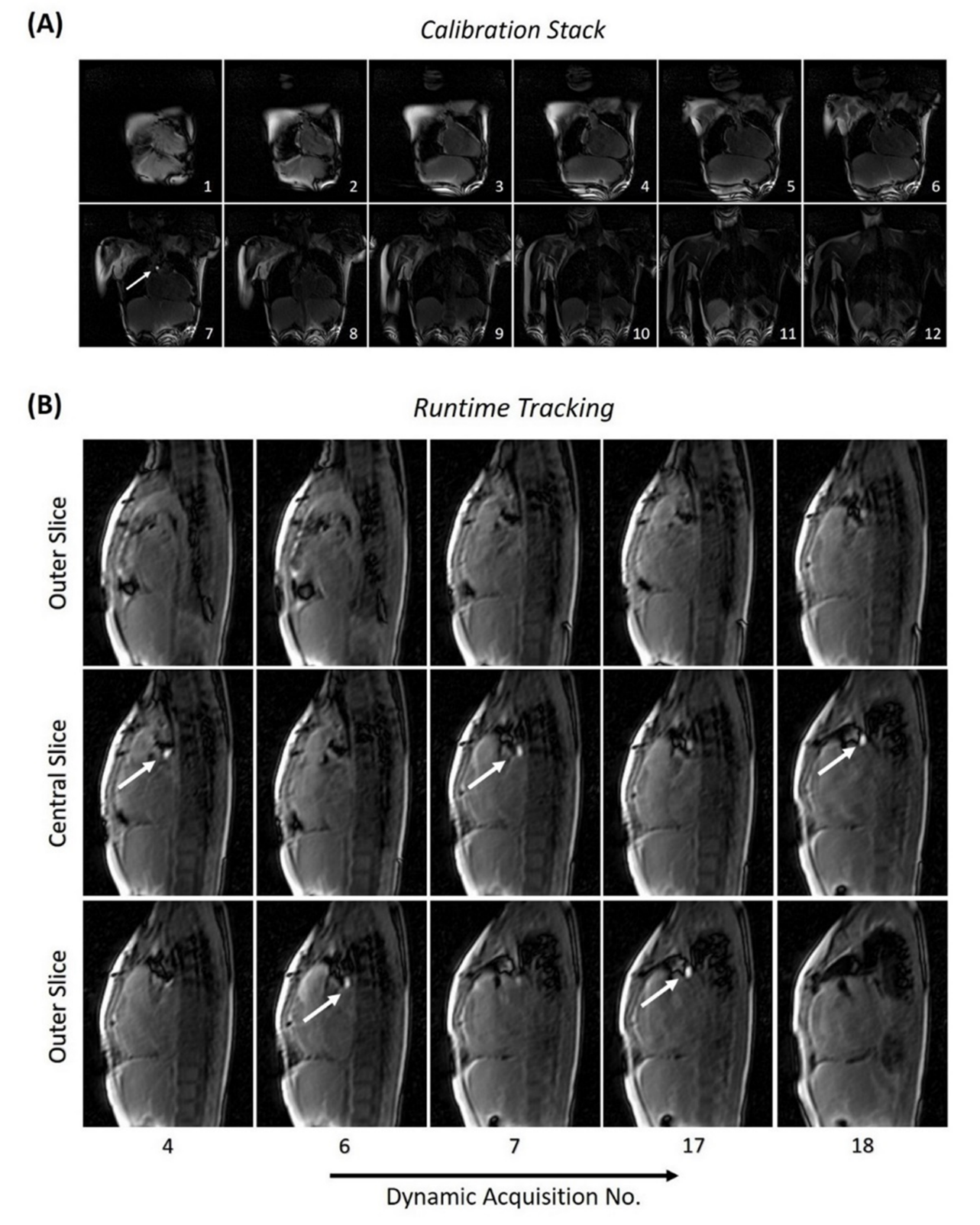


**Figure S1:** Representative images demonstrating the proposed approach in patient #1. (A) *Calibration* stack (12 slices) with the balloon initially identified in slice 7. (B) *Runtime* slices demonstrating automatic slice tracking and repositioning (between real-time measurements #6&7 and also #17&18) when the balloon was detected in one of the outer slices. The white arrows indicate the location of the automatically identified balloon.


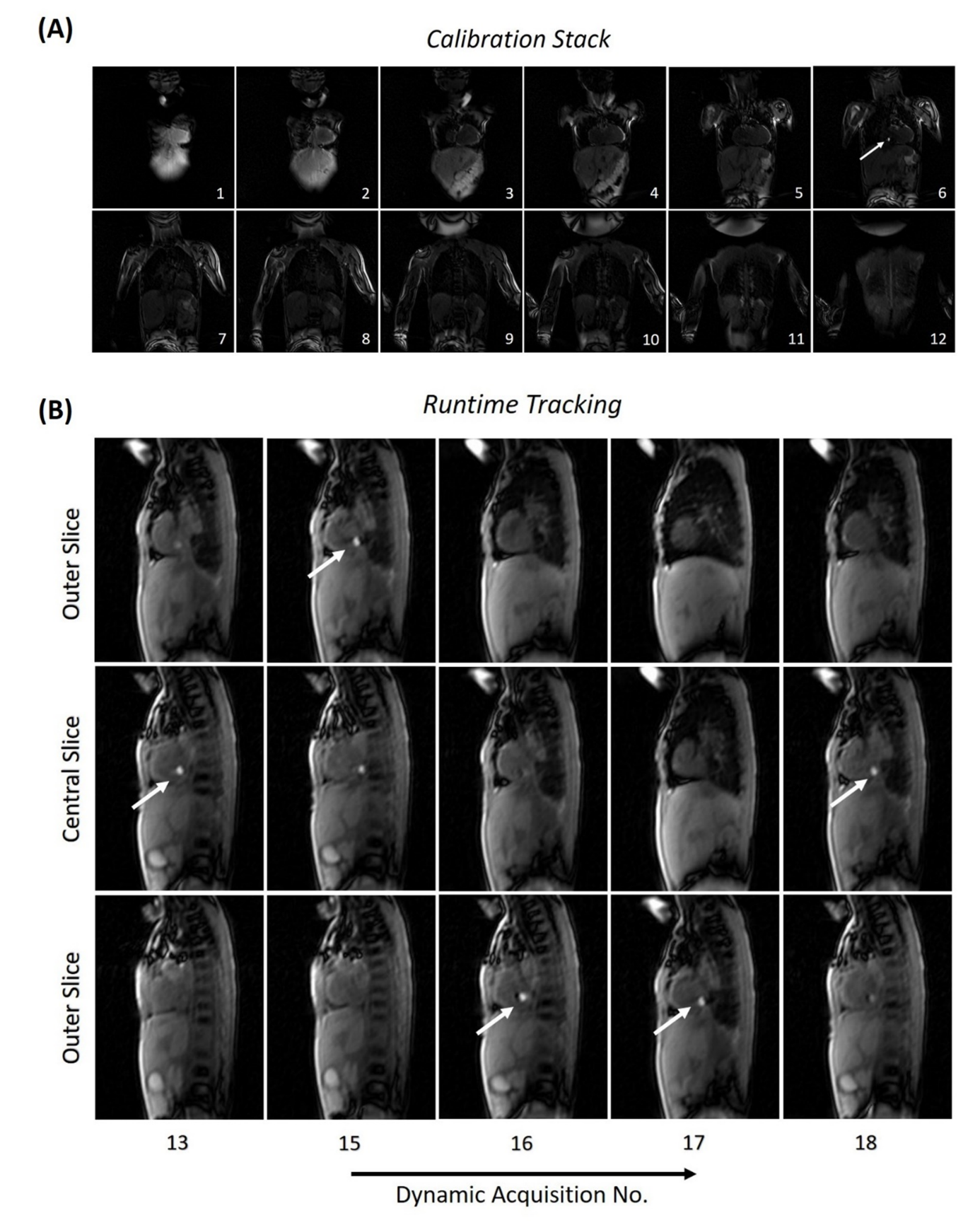


**Figure S2:** Illustration of the proposed approach in patient #2. The catheter balloon was again successfully identified in the *Calibration* stack (slice 6). During *Runtime*, the position of the three slices was automatically adjusted to ensure balloon visibility and follow the catheter in the central slice. The white arrows show the detected location of the catheter balloon.

**(4) Phantom experiment: single slice versus three slices in the *Runtime* mode**

The idea of having a single slice versus three slices in the *Runtime* mode was tested in a 3D-printed heart phantom, with the same acquisition parameters detailed in the Methods section of the main manuscript. The *Calibration* stack and *Runtime* slices were prescribed along the coronal orientation, and the same catheter trajectory was performed for the two experiments.

**Supplementary Information Videos S4** and **S5** show the results for the single-slice and three-slice experiments, respectively. The two videos show that, as expected, in the single-slice experiment, the sequence switches several times to the *Calibration* mode when the balloon falls out-of-plane during the *Runtime* mode. In comparison, in the three-slice experiment, for similar navigation of the catheter through the phantom by the interventionist, the balloon was always visible in one of the three slices in the *Runtime* mode. Furthermore, whenever the balloon was detected in one of the outer slices, slice repositioning was performed to keep the catheter in view in the central slice.

For the same catheter trajectory, the sequence switched from the *Runtime* to the *Calibration* mode seven times during the single-slice experiment, as compared to only one switch to *Calibration* during the three-slice experiment. Thus, this experiment demonstrates that the three-slice approach increases the in-plane time of the catheter balloon, making this approach particularly suitable for reducing the problem of out-of-plane catheters.
